# Comparative analysis of xanafide cytotoxicity in breast cancer cell lines

**DOI:** 10.1038/sj.bjc.6603829

**Published:** 2007-06-05

**Authors:** N Alami, J Paterson, S Belanger, S Juste, C K Grieshaber, B Leyland-Jones

**Affiliations:** 1 Department of Oncology, McGill University, 546 Pine Ave West, Montreal, QC, H2W 1S6 Canada; 2 Xanthus Pharmaceuticals Inc., 300 Technology Square, 5th Floor, Cambridge, MA 02139, USA

**Keywords:** breast cancer, xanafide, topoisomerase II, oestrogen receptor, p53

## Abstract

Xanafide, a DNA-intercalating agent and topoisomerase II inhibitor, has previously demonstrated comparable cytotoxicity to the parent drug amonafide (NSC 308847). The current study was conducted to investigate further the anti-proliferative effects of xanafide in human breast cancer cell lines, *in vitro* and *in vivo*. The *in vitro* activity of xanafide against MCF-7, MDA-MB-231, SKBR-3 and T47D cell lines was compared to that of paclitaxel, docetaxel, gemcitabine, vinorelbine and doxorubicin. In MCF-7, xanafide demonstrated comparable total growth inhibition (TGI) concentrations to the taxanes and lower TGI values than gemcitabine, vinorelbine and doxorubicin. MCF-7 (oestrogen receptor (ER)+/p53 wild-type) was the most sensitive cell line to xanafide. MDA-MB-231 and SKBR-3 exhibited similar sensitivity to xanafide. T47 D (ER+/p53 mutated), showed no response to this agent. The *in vivo* activity of xanafide was further compared to that of docetaxel in MCF-7 and MDA-MB-231 cell lines using the hollow fibre assay. Xanafide was slightly more potent than docetaxel, at its highest dose in MCF-7 cell line, whereas docetaxel was more effective than xanafide in MDA-MB-231 cell line. Our results show that there is no relationship between sensitivity of these cell lines to xanafide and cellular levels of both isoforms of topoisomerase II and suggest that ER and p53 status and their crosstalk may predict the responsiveness or resistance of breast cancer patients to xanafide.

Breast cancer is the most common malignancy affecting women in the Western world. Systemic chemotherapy is indicated for women with metastatic breast cancer (MBC) that is oestrogen/progesterone-negative or unresponsive to hormonal therapy, or rapidly progressing and life-threatening (Hortobagyi and Piccart-Gebhart, 1996). Several single-agent chemotherapeutic options have been shown to be effective as first- or second-line therapy in the management of MBC with the taxanes and anthracyclines being among the most active drugs (Pronzato and Rondini, 2006).

The taxanes, paclitaxel and docetaxel, bind to, and stabilise microtubules, causing cell-cycle arrest and apoptosis (Schiff *et al*, 1979). The overall response rates achieved in MBC patients were 29–63 and 47–65%, with paclitaxel and docetaxel, respectively (Montero *et al*, 2005). However, side effects such as hypersensitivity reactions, myelosuppression and neuropathy were observed with the taxanes, although docetaxel causes less neuropathy and more myelotoxicity (Ghersi *et al*, 2005).

Anthracyclines are active agents in primary adjuvant and palliative treatment of breast cancer (Kroger *et al*, 1999). Doxorubicin intercalates among DNA base pairs resulting in conformational changes in DNA structure and changes in the activity of topoisomerase. In MBC, response rates to single doxorubicin treatment range from 52% in previously untreated patients to 28% in patients previously exposed to an alkylating agent (Esteva *et al*, 2001). In patients who failed to respond to anthracycline and taxanes treatment, administration of capecitabine, gemcitabine or vinorelbine has been shown to result in overall response rates of 20–25% (Livingston *et al*, 1997; Blum *et al*, 1999; Valerio *et al*, 2001). Gemcitabine, an antimetabolite pyrimidine analogue, and vinorelbine, which inhibits microtubule polymerisation, have been widely investigated in the treatment of MBC (Martin *et al*, 2007).

In view of previous studies, in clinical practice, the potential therapeutic impact of standard chemo-agents for breast cancer is often limited due to intrinsic and/or acquired drug resistance in addition to drug-related toxicity. Therefore, balancing efficacy and toxicity is a major challenge, and the optimal choice of chemotherapy must be driven by patient and disease characteristics such as menopausal status, oestrogen and progesterone receptor status, Her-2 *neu* expression (Gralow, 2005), and tumour characteristics such as p53 gene involved in tumour response to therapy (Vogelstein *et al*, 2000).

Given the current status of the breast cancer treatment and to further improve the clinical outcome in these patients, new treatment approaches are needed.

Naphthalimides are DNA-intercalating agents that bind to DNA by insertion between the base pairs of the double helix. Intercalation causes the base pairs to separate vertically, thereby distorting the sugar-phosphate backbone and changing the degree of rotation between successive base pairs. Naphthalimides were designed by combining structural components of several antitumour compounds into a small molecule and have shown high antitumour activity upon a variety of murine and human tumour cells (Brana and Ramos, 2001). One of these compounds, amonafide (Sami *et al*, 1993) has been verified to exert ATP-independent topo II-mediated DNA cleavage (Andersson *et al*, 1987; Hsiang *et al*, 1989; Dorr *et al*, 2001). Furthermore, this agent is not affected by the multidrug resistance phenomenon and is not an efflux pump substrate (Dorr *et al*, 2001).

In previous clinical studies, amonafide demonstrated activity for the treatment of MBC (Costanza *et al*, 1995). However, it is metabolised to an active but myelosuppressive metabolite, the N-acetyl amonafide. A population study in women with breast cancer established that wide variation in the major side-effect of neutropenia was related to inter-individual patient differences in *N*-acetylating activity. Importantly, at the same prescribed dose, slow NAT2 acetylators were believed to be underdosed as reflected by minimal response rate and lack of grade 3 myelosuppression while rapid acetylators had greater haematologic toxicity attributed to slower clearance of the parent amonafide (Ratain *et al*, 1991, 1993). These data, and that from other studies in a variety of major tumour types, led to the conclusion that amonafide should not be considered for further clinical development.

Xanafide (amonafide L-malate), a novel salt formulation of amonafide, has been developed. Preliminary pharmacological and chemical equivalence studies demonstrated that xanafide had comparable pharmacokinetics and toxicity *in vitro* and *in vivo*, while having superior solubility, stability and compressibility than the parent drug, amonafide (personal communication). Individualising the dose of xanafide as a function of NAT2 phenotype may optimise patient safety and therapeutic benefit. Xanafide has recently received the FDA Orphan Drug Designation for the treatment of acute myeloid leukaemia (AML) and has now entered phase II clinical trials in patients with secondary AML.

The present study is the first one to report preclinical evaluation of xanafide. Our aim was to compare xanafide activity to standard breast cancer agents (paclitaxel, docetaxel, vinorelbine, gemcitabine and doxorubicin) in a panel of four breast cancer cell lines: MCF-7, MDA-MB-231, SKBR-3 and T47D, *in vitro*, and against paclitaxel in the first two cell lines, *in vivo*. This panel included oestrogen receptor (ER)-positive and ER-negative cell lines, since it has been shown that oestrogen can enhance the effects of topoisomerase II inhibitors in ER-responsive breast cell lines (Epstein and Smith, 1988).

## MATERIALS AND METHODS

### Cell lines

MCF-7, MDA-MB-231, SKBR-3 and T47D were obtained from the American Type Culture Collection (ATCC) (Rockville, MD, USA). Cell lines were grown in RPMI-1640 medium (Invitrogen, Gaithersburg, MD, USA), supplemented with 10% fetal bovine serum, 2 mM L-glutamine at 37°C in a humidified atmosphere containing 5% CO_2_.

### Chemicals and compounds

All general chemicals were purchased from Sigma Chemical Co. (St Louis, MO, USA) unless otherwise specified. Xanafide was kindly provided by Xanthus Life Sciences. Paclitaxel, docetaxel, doxorubicin, vinorelbine and gemcitabine are commercially available. Before *in vitro* use, a 1 mM stock solution of each agent was prepared by dilution in culture media. Polyvinylidene fluoride hollow fibres (500 000 Da molecular weight cutoff, 1.0 mm ID) were purchased from Spectrum Medical Industries (Luguan Hills, CA, USA).

### Animals

Athymic NCr *nu/nu* mice, 5–6 weeks of age were purchased from Taconic (Germantown, NY, USA). All studies involving these animals were conducted in accordance with National Cancer Institute (NCI) protocol and the McGill University Animal Care and Ethics guidelines.

### Cytotoxicity assay

Cytotoxicity studies were performed using the sulphorhodamine B assay (Skehan *et al*, 1990). Cytotoxicity of each drug was evaluated by the GI_50_ and TGI values, representing the 50% growth inhibition and total growth inhibition, respectively, compared to non-treated control (C) and a control at time of addition of increasing drug concentrations (Tz). On day 1, MCF-7 & SKBR-3 (5000 cells) and MDA-MB-231 & T47D (20 000 cells) were seeded into 96-well plates in a volume of 100 *μ*l per well. On day 2, one plate of each cell line was fixed *in situ* with trichloroacetic acid (TCA) to establish the cell population at time of drug addition (Tz). An aliquot of 100 *μ*l of serial dilutions of the different agents was added to the appropriate well, resulting in a series of final concentrations ranging from 0.1 nM to 100 *μ*M. After 48 h of drug exposure, the medium in control and drug-containing wells was removed. Cells were washed with cold phosphate-buffered saline (PBS) and then precipitated with 50 *μ*l ice-cold 50% TCA and fixed for 60 min at 4°. The supernatant was discarded, and cells were washed five times with tap water and air-dried. Fixed cells were then dyed with 50 *μ*l of 0.4% sulphorhodamine B in 1% acetic acid solution and the plates were incubated for 10 min at room temperature. Unbound dye was removed by washing with 1% acetic acid and the plates were air-dried. Sulphorhodamine B was dissolved in 150 *μ*l of 10 mM Tris-buffer (pH 10.5) and 540 nm optical density was measured in a Labsystems Multiskan® Multisoft apparatus. Percent net growth was calculated using the seven absorbance measurements (time zero, (Tz), growth control, (C), plus the test growth at the different drug concentration levels (Ti)) as follows: ((Ti–Tz)/(C–Tz)) × 100 for concentrations for which Ti>/=Tz and ((Ti–Tz)/Tz) × 100 for concentrations for which Ti<Tz. The results are expressed as the mean of three independent experiments±s.e.m.

### Evaluation of xanafide antitumour activity using the *in vivo* hollow fibre assay

The *in vivo* hollow fibre test was performed using the original NCI protocol (Hollingshead *et al*, 1995). Confluent monolayers of MCF-7 and MDA-MB-231 cells were harvested, collected by centrifugation and resuspended in conditioned medium. In preliminary NCI studies, cell growth was assessed with fibres containing various cell densities. As a result, cell densities of 2.5 × 10^6^ and 5 × 10^6^ cells ml^−1^ were found to be suitable for drug studies with MCF-7 and MDA-MB-231 cell lines, respectively. Fibres filled with cells at the respective densities were incubated in 6-well plates overnight at 37°C in a 5% CO_2_ atmosphere. Female athymic NCr *nu/nu* mice at 5–6 weeks of age were obtained from Taconic. Each mouse hosted up to six fibres, which were cultured in two physiological compartments. For intraperitoneal (i.p.) implants, a small incision was made through the skin and musculature of the dorsal abdominal wall, the fibre samples were inserted into the peritoneal cavity in a craniocaudal direction and the incision was closed with skin staples. For subcutaneous (s.c.) implants, a small skin incision was made at the nape of the neck to allow insertion of an 11-gauge tumour implant trocar. The trocar, containing the hollow fibre samples, was inserted caudally through the s.c. tissues and fibres were deposited during withdrawal of the trocar. The incision was closed with a skin staple.

Mice were randomised into three groups: saline control group (*n*=6), xanafide and docetaxel treated groups (*n*=3 for each group). Xanafide was dosed at the maximum tolerated dose 30 mg kg^−1^, on the basis of reported *in vivo* studies with amonafide (Dorr *et al*, 2001), and docetaxel at 5 and 12.5 mg kg^−1^ based on previous paclitaxel NCI tested doses (Hollingshead M, unpublished data). Both agents, in PBS were given once daily by i.p. injection from day 3–7 after implantation as reported previously (Hollingshead *et al*, 1995). Animals were monitored daily and clinical signs and body weights were recorded daily.

On day 8, mice were killed and fibres were retrieved. The fibres were placed into 6-well plates, with each well containing 2 ml of fresh, prewarmed culture medium and allowed to equilibrate for 30 min at 37°C. To define the viable cell mass contained within the intact hollow fibres, a 3-(4,5-dimethylthiazol-2-yl)-2,5-diphenyltetrazolium bromide (MTT) dye conversion assay was used. Briefly, 1 ml of prewarmed culture medium containing 1 mg MTT ml^−1^ was added to each dish. After incubating at 37°C for 4 h, the culture medium was aspirated and the samples were washed twice with normal saline containing 2.5% protamine sulphate solution followed by overnight incubation at 4°C. To assess the optical density of the samples, the fibres were transferred to 24-well plates, cut in half and allowed to dry overnight. The formazan was extracted from each sample with dimethylsulphoxide (250 *μ*l well^−1^) for 4 h at room temperature on a rotation platform. Aliquots (150 *μ*l) of extracted MTT formazan were transferred to individual wells of 96-well plates and assessed for optical density at a wavelength of 540 nm. Results are expressed as % growth inhibition compared to control±s.d.

### Statistical analysis

The comparisons between the untreated and treated groups were analyzed using the Student's *t*-test. Two-sided *P*-values less than 0.05 were considered statistically significant.

## RESULTS

### *In vitro* antiproliferative activity of xanafide in human breast cancer cells

A panel of four human breast cancer cell lines: MCF-7, MDA-MB-231, SKBR-3 and T47D was used in this study. Their molecular characteristics are listed in Table 1. Using the SRB assay, the cytotoxicity profile of xanafide was compared with those of five anticancer drugs widely used in the clinic: paclitaxel, docetaxel, doxorubicin, gemcitabine and vinorelbine. The results were expressed as GI50 and TGI values and summarised in Table 2.

**Figure 1:**
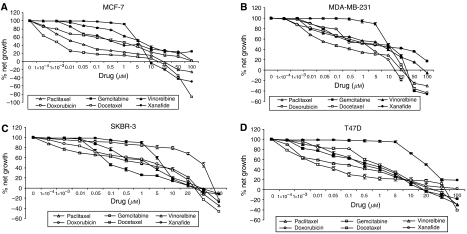
*In vitro* cytotoxicity of xanafide in breast cell lines in comparison with common drugs. (**A**) MCF-7, (**B**) MDA-MB-231, (**C**) SKBR-3, (**D**) T47D. Xanafide (•), vinorelbine (▴), gemcitabine (▪), doxorubicin (○), paclitaxel (▵) and docetaxel (□). Cells were exposed to the different drugs for 48 h. % Net growth was determined with the SRB assay as described in Materials and Methods. The results are the average of three separate experiments±s.e.m.

After 48 h exposure time, xanafide demonstrated a steep response curve in the four breast cell lines tested (Figure 1A–D). Xanafide inhibited the growth of the ER-positive MCF-7 and T47D cells in a concentration-dependent manner, with an average GI_50_ value of 5 and 20 *μ*M, respectively. Xanafide also inhibited the growth of the ER-negative SKBR-3 and MDA-MB-231 cells in a concentration-dependent manner, with an average GI_50_ value of 6 and 10 *μ*M, respectively (Table 2). T47 D was the less sensitive to the antiproliferative effect of xanafide (Figure 1D). Notably, no total growth inhibition of T47D was obtained which could, in part, be due to the long doubling time of this cell line (45.5 h).

To better visualise the differences in the cytotoxicity to achieve complete cell growth inhibition for all the agents, TGI concentrations (resulting in total growth inhibition) were expressed. At doses higher than the TGIs, net cell killing was observed in the four breast cell lines tested: MCF-7, MDA-MB-231, SKBR-3 and T47D (Table 2).

In MCF-7 cell line, xanafide exhibited a 1.7–2.2-fold lower TGI concentration than those of docetaxel and paclitaxel, respectively. Vinorelbine and doxorubicin induced similar effects and their TGI concentrations were 10-fold higher than that of xanafide. No total growth inhibition was achieved with gemcitabine (Table 2).

In MDA-MB-231 cell line, doxorubicin induced total growth inhibition at the lowest concentrations: 15 *μ*M, whereas paclitaxel, docetaxel and xanafide exhibited comparable TGI values: 20, 25 and 35 *μ*M. Vinorelbine and gemcitabine were less potent as shown by their respective 2.6- to 5.7-fold higher TGI concentrations, respectively (Table 2).

In SKBR-3 cells, gemcitabine and docetaxel induced total growth inhibition at 30 *μ*M. Paclitaxel, xanafide and vinorelbine exhibited comparable TGI concentrations, 35, 45 and 50 *μ*M, respectively. The TGI for doxorubicin was 80 *μ*M, 9-fold higher than that of xanafide (Table 2).

In T47D cell line, vinorelbine and gemcitabine induced similar cytotoxicity (TGI concentration of 17 *μ*M). Paclitaxel and docetaxel showed TGI concentrations of 35 and 60 *μ*M, respectively. Xanafide did not induce any complete growth inhibition in this cell line (Table 2).

These results indicate that the four breast cell lines tested exhibited differential sensitivity to xanafide and the common chemotherapeutic agents tested. Considering the TGI concentrations (Table 2) and the net cell killing achieved by xanafide, MCF-7 was the most sensitive cell line; MDA-MB-231 and SKBR-3 were almost equally sensitive while T47D was less responsive to this agent.

### *In vivo* antitumour activity

On the basis of its cytotoxic activity *in vitro*, xanafide was further evaluated for *in vivo* activity in two ER+ and ER− breast cancer cell lines, MCF-7 and MDA-MB-231, respectively. The two cell lines were implanted i.p. and s.c. in NCr nude mice using the hollow fibre assay. Animals were treated with saline (control group), docetaxel dosed at 5 and 12.5 mg kg^−1^, i.p., or xanafide at 30 mg kg^−1^, i.p. on a q.d. × 5 treatment schedule, beginning on day 2 post-fibre implantation (days 3–7).

**Figure 2:**
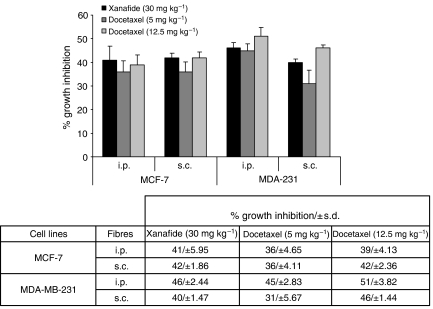
Antitumour effect of xanafide in comparison with docetaxel in MCF-7 and MDA-MB-231 breast cell lines in the *in vivo* hollow fibre assay. Fibres filled with the cells were implanted at the intraperitoneal (i.p.) and subcutaneous (s.c.) compartments of NCr *nu/nu* mice. The animals were treated with saline (control), xanafide (30 mg kg^−1^) or docetaxel (5 & 12.5 mg kg^−1^). Drugs were administrated once daily by i.p. injection from days 3–7 after implantation. On day 8, mice were killed and fibres were retrieved. The effectiveness of the drugs was evaluated on the basis of growth inhibition of the cells determined by MTT assay, as described in Materials and Methods.

As shown in Figure 2, in the fibres retrieved from the i.p. sites, xanafide, administered as single agent, was effective at reducing the tumour cells growth of MCF-7 and MDA-MB-231 by 41 and 46%, respectively, as compared with control (*P*<0.05). Docetaxel exhibited dose-dependent growth inhibitory effects. At the lowest dose used, 5 mg kg^−1^, docetaxel produced a growth inhibition of 36 and 45%, in MCF-7 and MDA-MB-231, respectively (*P*<0.05). At 12.5 mg kg^−1^, the growth inhibition elicited was 39 and 51%, in MCF-7 and MDA-MB-231, respectively (*P*<0.05). These results show that xanafide was slightly more potent than docetaxel at its highest dose, in MCF-7. In contrast, its potency was lower than that of docetaxel in MDA-MB-231.

In the fibres retrieved from the s.c. sites (Figure 2), xanafide administered as single agent produced comparable growth inhibition in MCF-7 and MDA-MB-231 cell lines, reducing the growth by 42 and 40%, respectively (*P*<0.05). Docetaxel showed dose-dependent inhibitory effects. At the lowest dose, the growth inhibition obtained was 31 and 36%, in MDA-MB-231 and MCF-7, respectively (*P*<0.05). At the highest dose used, 12.5 mg kg^−1^, the growth inhibition induced was 42 and 46%, in MCF-7 and MDA-MB-231, respectively (*P*<0.05).

**Figure 3:**
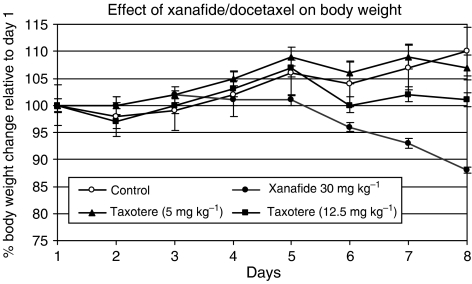
Relative body weight change. Mice were implanted with i.p. and s.c. fibres filled with MCF-7 and MDA-MB-231 breast cell lines. Mice were treated with vehicle (—○—), xanafide (—•—), docetaxel (5 mg kg^−1)^ (—▴—) and docetaxel (12.5 mg kg^−1^) (—▪—).

Body weights were recorded daily during the course of the study, and expressed as the difference observed relative to start of treatment. For the docetaxel treated mice no loss in body weight was observed while in the xanafide treated mice, body weight was reduced by 12% by day 8, which was within the acceptable range based on NCI established criteria (Figure 3).

## DISCUSSION

Amonafide, a DNA-intercalating agent and topoisomerase II inhibitor, has been used as first-line treatment for MBC. However, amonafide is extensively metabolised, including N-acetylation to an active metabolite, N-acetyl amonafide, and the extent of amonafide N-acetylation is the major determinant of myelosuppression (Ratain *et al*, 1995). As a result, several compounds having structural analogy to amonafide have been synthesized (Sami *et al*, 1993, 2000; Dorr *et al*, 2001). Among these, azonafide has shown high potency against a panel of human colon cancer cell lines and was active against i.p. P388 leukaemia and s.c. B16 melanoma murine models (Sami *et al*, 1993).

Xanafide, the new formulation of amonafide, has been synthesized aiming at reducing the toxicity and improving the therapeutic index of the parent drug, amonafide. We have previously shown that xanafide and amonafide hydrochloride have comparable and significant inhibitory activity both *in vitro* against the three cell lines of the NCI prescreen program: H460 (non-small cell lung), SF268 (glioma) and MCF-7 (breast), and *in vivo* in MCF-7 (breast), COLO205 (colon) and PC-3 (prostate) cell lines, using the hollow fibre assay (Alami *et al*, 2003).

The aim of this study was to further investigate the antitumour effects of xanafide, in comparison with common breast drugs in a panel of breast cell lines well characterised for their oestrogen receptor, p53 status, Her-2 and topoisomerase II *α* and *β* levels: MCF-7, MDA-MB-231, SKBR-3 and T47D (Table 1). Xanafide exhibited a steep response curve in all four cell lines tested (Figure 1A–D). The GI 50 and TGI concentrations clearly show that these cell lines displayed differential sensitivity to xanafide with MCF-7 (ER^+^/p53 wild-type) being the most sensitive and T47 D (ER+/p53 mutated) the most resistant. Although this cell line was responsive to low doses of the other drugs tested, the lack of activity of xanafide in the T47D could be, in part, due to its long doubling time.

Furthermore, xanafide has proved to be more active than the taxanes, gemcitabine, vinorelbine and doxorubicin in MCF-7. The Two ER^−^/p53 mutated cell lines (MDA-MB-231 and SKBR-3) displayed comparable *in vitro* responsiveness to xanafide as demonstrated by their respective GI50 and TGI concentrations (Table 2).

Our *in vitro* results correlate with the *in vivo* data where xanafide was slightly more potent than docetaxel at its highest dose, in MCF-7 (Figure 2). These findings suggest a specificity of xanafide against the ER^+^, p53 w/t MCF-7 cell line.

These data raise the question of what are the mechanisms underlying the response to xanafide. Several clinical observations indicate a role for oestrogen and ER in the development, progression and treatment of human breast cancer (Angeloni *et al*, 2004). In addition, there is substantial evidence showing that alterations in the tumour suppressor gene, p53, are associated with the development of several types of cancer, including breast cancer (Hollstein *et al*, 1991). Considering that the p53 gene is mutated in approximately 50% of all tumours, its role in the control of cell cycle progression, maintenance of DNA integrity and induction of apoptosis is well documented (Zhan *et al*, 1993; Yonish-Rouach *et al*, 1995). It has also been shown that in breast cancer, p53 mutations are associated with a decrease in disease-free and overall survival of patients (Sirvent *et al*, 2001). Previous studies have reported that the ability of p53 to control the expression of ER*α* could suggest that specific p53 mutations in breast tumours may contribute not only to oncogenesis and drug resistance, but also to the more aggressive phenotype associated with the loss of ER expression. Interestingly, a high percentage of breast tumours with p53 mutations are ER-negative (Berns *et al*, 1996).

Our results showed that the two ER^−^/p53 mutated cell lines MDA-MB-231 and SKBR-3 exhibited moderate sensitivity to xanafide, whereas T47 D (ER+/p53 mutated) was more resistant to xanafide, with no induced cell killing, as compared with the extent of the anti-proliferative effect observed with MCF-7 (ER+/p53 wt) (Table 2). These findings may partially support a mechanism of responsiveness to xanafide in ER+ breast cancer where an active p53 gene is required. Additionally, related studies have shown that p53 was a negative regulator of the ER signalling pathways, suggesting a crosstalk between p53 and ER in breast cancer (Yu *et al*, 1997).

On the other hand, the DNA damage induced by topo II inhibitors triggers the p53-dependent apoptotic pathway that lead to cell cycle arrest (p21, cyclins, GADD45, PCNA) or to apotosis (Bcl-2, Bax, surviving, scotin) (Valkov and Sullivan, 2003). Related studies using tumour cell lines tested for their p53 status have shown that mutations of p53 correlate with drug resistance to a wide spectrum of anticancer agents, including topo II inhibitors (O'Connor *et al*, 1997; Sirvent *et al*, 2001). Usually, wild-type p53 expression predisposes cells to a more rapid rate of cell death after DNA damage. Previous studies have reported that non-small cell lung cancer cells having p53 mutations showed significantly poorer response to intensive chemotherapy that included etoposide and epirubicin (Vogt *et al*, 2002). In addition, treatment of MCF-7 with doxorubicin resulted in an increase of p53 expression, confirming a p53-mediated response to doxorubicin in cells containing a wild-type p53 gene product (Liem *et al*, 2003). Additional work has revealed that loss of wild-type p53 function confers resistance to etoposide in neuroblastoma cells (Keshelava *et al*, 2001) and in glioma cells (Yin *et al*, 2000). Others have also reported that p53 status in breast cancer patients correlated with a poor response to epirubicin (Mottolese *et al*, 2000). Furthermore, it has been postulated that the cell cycle arrest effect of wild-type p53 on cell cycle regulated topo II*α* expression could potentially provide a sufficient amount of target enzyme for optimisation of treatment with topoisomerase II inhibitors (Valkov and Sullivan, 2003), thus supporting the greater potency of xanafide in MCF-7 cell line.

In addition, the topoisomerase II *α* gene, TOP2 *α*, is located at chromosome band 17q12-q21, close to the ErbB-2 oncogene (HER-2/neu), which is the most commonly amplified oncogene in breast cancer. Because of the physical proximity to ErbB-2, copy number aberrations may also occur in the topoII*α* gene, which may be related to the altered chemosensitivity to topoII inhibitors (Jarvinen and Liu, 2006). Our results show that xanafide sensitivity was not correlated with expression levels of both topoisomerase II *α* and *β*. Considering the TGI concentrations, MDA-MB-231 and SKBR-3 expressing low levels and high levels of both isoforms, respectively, exhibited comparable sensitivity to xanafide. Our data are in agreement with those reported by Tewey *et al* (1984) where sensitivity to the topo II inhibitor, doxorubicin, showed no correlation with expression of either of the topoisomerase II isoforms.

In view of the previous findings together with our results, we speculate that p53 and ER and their signalling pathways are likely important determinants of breast tumour cells sensitivity to xanafide. Understanding these relationships may lead to strategies for xanafide-based chemotherapy optimisation and further precision targeting of tumour cells to avoid drug resistance and thereby chemotherapy failure. Furthermore, the steep response curves of xanafide activity in the four breast cell lines tested suggest that accurate individualised patient dosing is critically important for maximising clinical response while minimising neutropenic variability.

## Figures and Tables

**Table 1:** Characteristics of breast cell lines

				**Topo-II levels (absorbance)^a^**	
**Cell line**	**ER status**	**p53 status**	**Her levels**	***α***	***β***
MCF-7	+	*wt*	Very low	1086	1233
MDA-MB-231	−	*mu*	Low	724	304
SKBR-3	−	*mu*	High	1656 (amplified)	1202 (amplified)
T47D	+	*mu*	Moderate	588	1188

Abbreviation: ER=oestrogen receptor.

a Houlbrook *et al* (1995).

**Table 2:** *In vitro* profile of xanafide in comparison with common drugs

	**GI50/TGI±s.e.m. (*μ*M)**							
	**MCF-7**		**MDA-MB-231**		**SKBR-3**		**T47D**	
Xanafide	5±1.1	9±1.8	10±1.5	35±5.1	6±0.9	45±5.3	20±2.2	—
Paclitaxel	0.01±0.004	20±1.9	0.1±0.04	20±2.1	1±0.3	35±4.3	0.1±0.02	35±3.7
Docetaxel	0.001±0.0005	15±1.8	5±0.9	25±2.8	5±1.0	30±4.1	0.01±0.006	60±5.5
Gemcitabine	0.5±0.04	—	1±0.4	>100	0.1±0.03	30±4.0	0.5±0.07	17±2.3
Vinorelbine	5±1.3	100±10.2	5±0.7	90±	2±0.3	50±5.6	0.5±0.05	17±2.2
Doxorubicin	0.5±0.06	100±18.3	0.5±0.003	15±1.8	40±3.8	80±7.8	1±0.3	100±

GI50 and TGI concentrations calculated after 48 h exposure. Results are mean of three independent experiments. GI50 was calculated as the drug concentration that reduced the number of cells to 50% of the number of cells before drug addition. TGI is the drug concentration that achieved total growth inhibition of the cells.
